# Curated compendium of human transcriptional biomarker data

**DOI:** 10.1038/sdata.2018.66

**Published:** 2018-04-17

**Authors:** Nathan P. Golightly, Avery Bell, Anna I. Bischoff, Parker D. Hollingsworth, Stephen R. Piccolo

**Affiliations:** 1 Department of Biology, Brigham Young University, Provo, Utah 84602, USA; 2 Northeast Ohio Medical University, Rootstown, Ohio 44272, USA; 3 Department of Biomedical Informatics, University of Utah, Salt Lake City, Utah 84602, USA

**Keywords:** Data publication and archiving, Prognostic markers, Predictive markers, Diagnostic markers, Cancer

## Abstract

One important use of genome-wide transcriptional profiles is to identify relationships between transcription levels and patient outcomes. These translational insights can guide the development of biomarkers for clinical application. Data from thousands of translational-biomarker studies have been deposited in public repositories, enabling reuse. However, data-reuse efforts require considerable time and expertise because transcriptional data are generated using heterogeneous profiling technologies, preprocessed using diverse normalization procedures, and annotated in non-standard ways. To address this problem, we curated 45 publicly available, translational-biomarker datasets from a variety of human diseases. To increase the data's utility, we reprocessed the raw expression data using a uniform computational pipeline, addressed quality-control problems, mapped the clinical annotations to a controlled vocabulary, and prepared consistently structured, analysis-ready data files. These data, along with scripts we used to prepare the data, are available in a public repository. We believe these data will be particularly useful to researchers seeking to perform benchmarking studies—for example, to compare and optimize machine-learning algorithms' ability to predict biomedical outcomes.

## Background & Summary

DNA encodes a cell’s instruction manual in the form of genes and regulatory sequences^1^. Cells behave differently, in part, because genes are transcribed into RNA in different quantities within those cells^2^. Researchers examine gene-expression levels to understand cellular dynamics and the mechanisms behind cellular aberrations, including those that lead to disease development. Modern technologies now make it possible to profile expression levels for thousands of genes at a time for a modest expense^3^. Using these high-throughput technologies, scientists have performed thousands of studies to characterize biological processes and to evaluate the potential for precision-medicine applications. One such application is to derive *transcriptional biomarkers*—patterns of expression that indicate disease states or that predict medical outcomes, such as relapse, survival, or treatment response^4–10^. Indeed, already to date, more than 100 transcriptional biomarkers have been proposed for predicting breast-cancer survival alone^11^.

Many funding agencies and academic journals have imposed policies that require scientists to deposit transcriptional data in publicly accessible databases. These policies seek to ensure that other scientists can verify the original study's findings and can reuse the data in secondary analyses. For example, Gene Expression Omnibus (GEO) currently contains data for more than 2 million biological samples^12^. Upon considering infrastructure and personnel costs, we estimate that these data represent hundreds of millions—if not billions—of dollars (USD) of collective research investment. Reusing these vast resources offers an opportunity to reap a greater return on investment—perhaps most importantly via informing and validating new studies. Unfortunately, although anyone can access GEO data, researchers vastly underutilize this treasure trove because preparing data for new analyses requires considerable background knowledge and informatics expertise.

In GEO, data are typically available in two forms: 1) raw data, as produced originally by the data-generating technology, and 2) processed data, which were used in the data generators' analyses. In most cases, researchers process raw data in a series of steps that might include quality-control filtering, noise reduction, standardization, and summarization (e.g., summarizing to gene-level values and excluding outliers). Data from different profiling technologies must be handled in ways that are specific to each technology. However, even for datasets generated using the same profiling technology, the methods employed for data preprocessing vary widely across studies. This heterogeneity makes it difficult for researchers to perform secondary analyses and to trust that analytical findings are driven primarily by biological mechanisms rather than differences in data preprocessing. In addition, when data have not been mapped to biologically meaningful identifiers, it may be difficult for researchers to draw biological conclusions from the data.

Sample-level annotations accompany each GEO dataset. For biomarker studies, such metadata might include medical diagnoses or treatment outcomes, as well as covariates such as age, sex, or ethnicity. Although GEO publishes metadata in a semi-standardized format and bioinformatics tools exist for downloading and parsing GEO data^13,14^, it is difficult for many researchers to extract these data into a form that is suitable for secondary analyses. Within annotation files, values are often stored in key/value pairs with nondescript column names. Many columns are not useful for analytical purposes (e.g., when all samples have the same value). When values are missing, the columns often become shifted; accordingly, data for a given variable may be spread across multiple columns. Moreover, a variety of descriptors (e.g., “?”, “N/A”, or “Unknown”) are used to indicate missing values, thus requiring the analyst to account for these differences. In addition, seemingly minor errors, such as spelling mistakes or inconsistent capitalization, can hamper secondary-analysis efforts.

**Figure 1:**
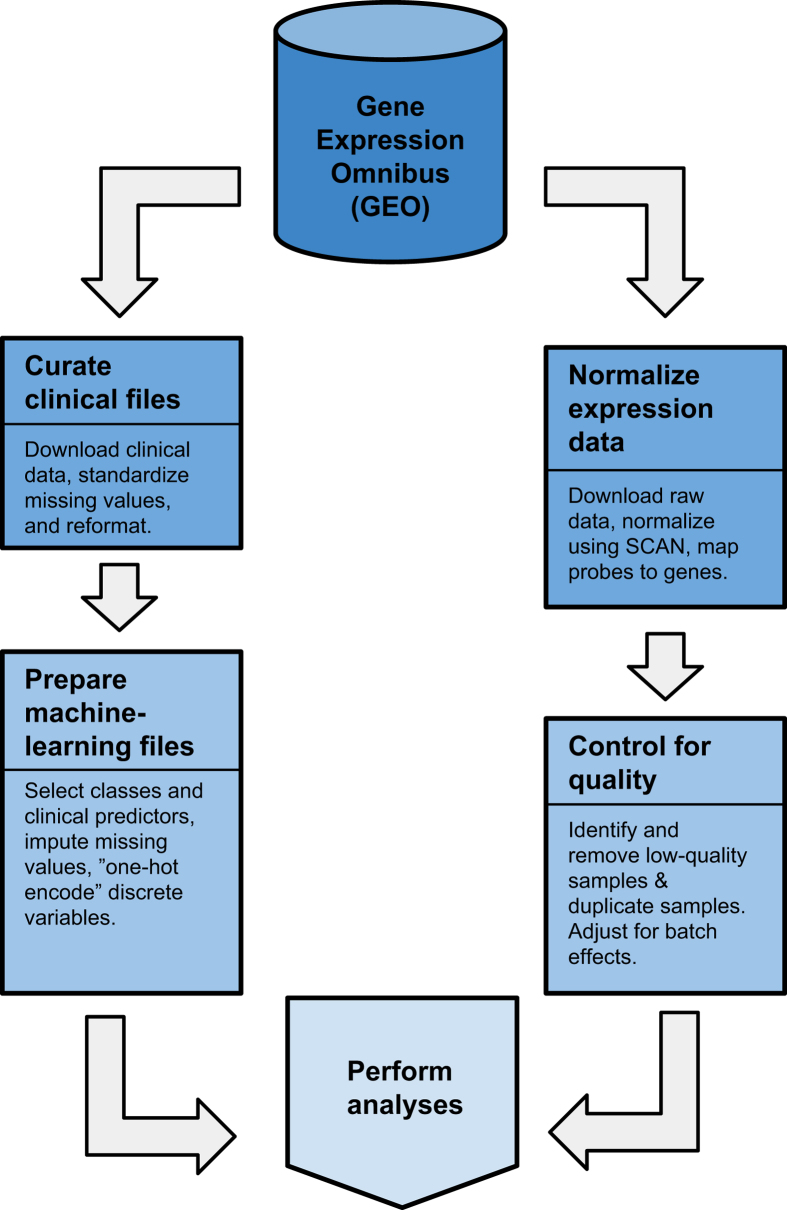
Flow diagram that illustrates the process we used to collect and curate the data. We wrote computer scripts that downloaded the data, checked for quality, normalized and standardized data values, and stored the data in analysis-ready file formats. The specific steps differed for clinical and expression data (see Methods).

In response to these challenges, we compiled the *Biomarker Benchmark*, a curated compendium of 45 transcriptional-biomarker datasets from GEO. These datasets represent a variety of human-disease states and outcomes, many related to cancer. We obtained raw gene-expression files, renormalized them using a common algorithm, and summarized the data using gene-level annotations (Figure 1). We used two techniques to check for quality-control issues in the gene-expression data. For datasets where gene-expression data were processed in multiple batches—and where batch information was available—we corrected for batch effects. Finally, we prepared a version of the data that is suitable for direct application in machine-learning analyses. For this version of the data, we one-hot encoded any discrete values and imputed any missing values.

## Methods

### Selecting data

To select datasets to be included in our compendium, we performed a custom search in Gene Expression Omnibus (GEO). First, we limited our search to data series that were associated with the Medical Subject Heading (MeSH) term "biomarker" and that came from *Homo sapiens* subjects. Next we limited the search to data generated using Affymetrix gene-expression microarrays and for which raw expression data were available (so we could renormalize the data). For each dataset, we examined the metadata to ensure that each series had at least one biomarker-relevant clinical variable. These included variables such as prognosis, disease stage, histology, and treatment success or relapse. Lastly, we selected series that included data for at least 70 samples (before additional filtering, see below).

Based on these criteria, we identified 36 GEO series. Two series (GSE6532 and GSE26682, Data Citation 1) contained data for two types of Affymetrix microarray. To avoid platform-related biases, we separated each of these series into two datasets; we used a suffix for each that indicates the microarray platform (e.g., GSE6532_U133A and GSE6532_U133Plus2). For both of these series, the biological samples profiled using either microarray platform were distinct. The GSE2109 series—known as the Expression Project for Oncology (expO)—had been produced by the International Genomics Consortium and contains data for 129 different cancer types^15^. To avoid confounding effects due to tissue-specific expression and because the metadata differed considerably across the cancer types, we split this dataset into multiple datasets based on cancer type (Table 1 (available online only)). We excluded tissue types for which fewer than 70 samples were available; we also excluded the "omentum" cancer type because it was relatively heterogeneous and had relatively few samples.

We used publicly available data for this study and played no role in contacting the research subjects. We received approval to work with these data from Brigham Young University's Institutional Review Board (E 14522).

### Preparing clinical annotations

For each dataset, we wrote custom R scripts^16^ that download, parse, and reformat the clinical annotations. Initially, these scripts retrieve data from GEO using the *GEOquery* package^13^. Next they generate a tab-delimited text file for each dataset that contains all available clinical annotations, except those with identical values for all samples (for example, platform name, species name, submission date) or that were unique to each biological sample (for example, sample title). In addition, these scripts generate Markdown files that summarize each dataset and indicate sources.

**Figure 2:**
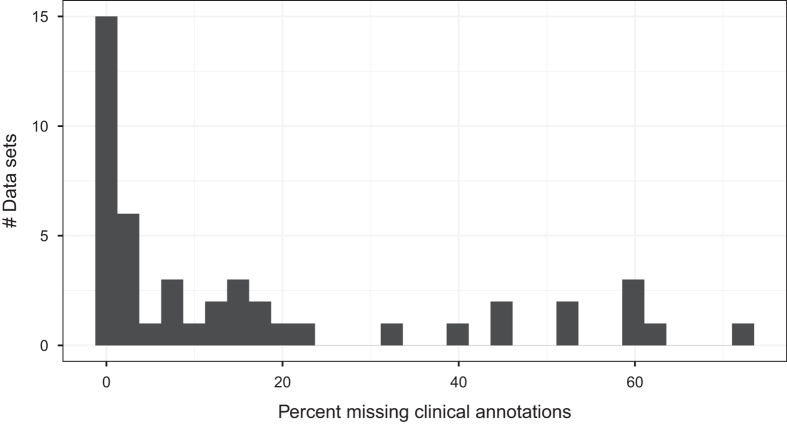
Histogram showing the proportion of missing clinical-annotation values per dataset. Some datasets contained no missing values, while others were missing as many as as 72.3% of data values.

In some cases, multiple data values are included in the same cell in GEO annotation files. For example, in GSE5462 (Data Citation 1), one patient's clinical demographics and treatment responses are listed as "female; breast tumor; Letrozole, 2.5 mg/day,oral, 10–14 days; responder." We parsed these values and split them into separate columns for each sample. After these cleaning steps, the datasets contained an average of 7.8 variables of metadata (Table 1 (available online only)). Next we searched each dataset for missing values. Across the datasets, 11 distinct expressions had been used by the original data generators to represent missingness; these included "N/A", "NA", "MISSING", "NOT AVAILABLE", "?", and others. To support consistency, we standardized these values across the datasets, using a value of "NA". On average, 17.0% of the metadata values were missing per dataset; this proportion differed considerably across the datasets (Figure 2).

We anticipate that many researchers will use these data to develop and benchmark machine-learning algorithms (although they can be used in many other types of analysis). Accordingly, we prepared a secondary version of the clinical annotations that are ready to use in machine-learning analyses. First, we identified class variables that have potential relevance for biomarker applications. In many cases, these variables were identical to those used in the original studies; but we also included class variables that had not been used in the original studies. On average, the datasets contain 2.8 class variables. Second, we identified clinical variables that could be used as predictor variables (covariates). Using these data, we generated one "Analysis" file per class variable that contains the class values for each sample as well as covariates that we suggest are relevant to the class variable. (A given variable may be used as a class variable in one context and a predictor variable in a different context.) We named these analysis files using descriptive prefixes (e.g., "Prognosis", "Diagnosis", or "Stage"). In addition, we identified concepts in the National Cancer Institute Thesaurus^17^ that map to each class and covariate variable. The name of each analysis file indicates the thesaurus term (preferred name) that corresponds to the class variable for that file. Within these files, the column names indicate the thesaurus terms that correspond to each covariate. We hope the use of this controlled vocabulary will make it easier for others to better understand the semantic meaning of these variables and identify commonalities across datasets. A tab-separated file that indicates mappings between the original annotation terms and the thesaurus terms can be found in our data repository (see https://osf.io/szwx6/).

When a given sample was missing data for a given class variable, we excluded that sample from the respective analysis file for that class variable. After this filtering step, we identified class variables with fewer than 40 samples and excluded these class variables. When covariates were missing more than 20% data (Figure 2), we excluded these variables from the analysis files. When covariates were missing less than 20% data, we imputed missing values using median-based imputation for continuous variables and mode-based imputation for discrete variables^18^. We transformed discrete predictor variables using one-hot encoding; each unique value, except the first, was treated as a binary variable. In cases where discrete values were rare, we merged values. For example, in GSE2109_Breast (Data Citation 1), we merged *Pathological_Stage* values 3A, 3B, 3C, and 4 into a category called "3-4" because relatively few patients fell into the individual categories (38, 8, 22, and 5 samples, respectively). In addition, some class variables were ordinal in nature (e.g., cancer stage or tumor grade); we transformed these into binary variables. Finally, some clinical outcomes were survival or relapse times; we transformed these data to (discrete) class variables, using dataset-specific thresholds to distinguish between "long-term" and "short-term" survivors and excluding patients who were censored after the survival threshold had been reached. Our computer scripts (see Code availability) encode these decisions for each dataset.

### Preprocessing gene-expression data

We created a computational pipeline (using R and shell scripts) that downloads, normalizes, and standardizes the raw-expression data. We used the *GEOquery* package^13^ to download the CEL files and then normalized them using the *SCAN.UPC* package^19^. Some heterogeneity exists, even among platforms from the same manufacturer (Affymetrix). The number of probes and the probe sequences used in designing the microarray architectures vary. To help mitigate this heterogeneity and to aid in biological interpretation, we summarized the data using Ensembl-based gene-level annotations from *Brainarray*^20,21^. The SCAN algorithm log2-transforms the data and scales the data to center around zero. Relatively high values indicate relatively high gene-expression levels, and vice versa.

### Code availability

Our computer scripts are stored in the open-access *Biomarker Benchmark* repository (Data Citation 1). Using these scripts, other researchers can reproduce our curation process and/or produce alternative versions of the data.

## Data Records

After we filtered the original data (see Methods), our compendium includes data for 7,037 biological samples across 45 datasets (Table 1 (available online only)). On average, the datasets contain values for 18,043 genes (Table 1 (available online only)). In total, our repository contains 128 class variables (2.8 per dataset) and 2.1 unique values per class variable.

All output data are stored in tab-delimited text files and are structured using the "tidy data" methodology^22^. Accordingly, data users can import the files directly into analytical tools such as Microsoft Excel, R, or Python. All data files are publicly and freely available in the open-access *Biomarker Benchmark* repository (Data Citation 1). The original data files are available via Gene Expression Omnibus using the accession numbers listed in Table 1 (available online only).

## Technical Validation

**Figure 3:**
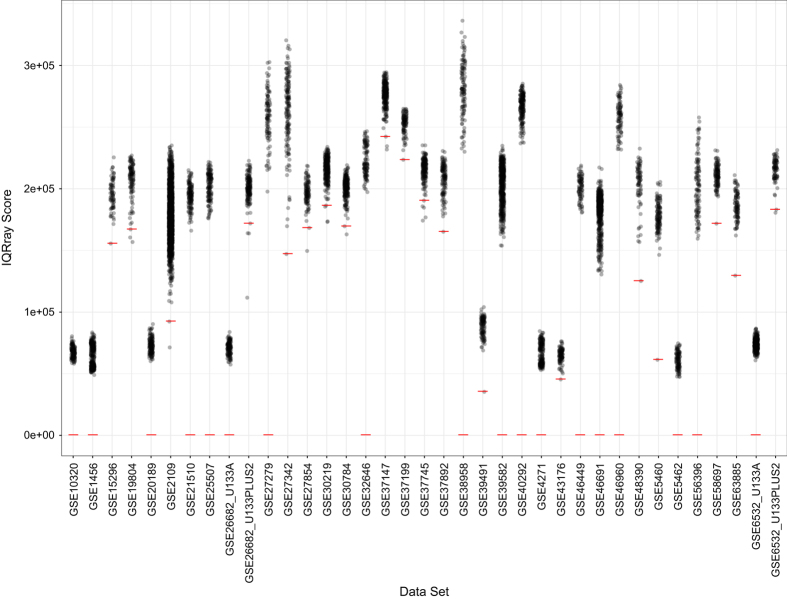
Distribution of IQRay quality scores for each dataset. Sample qualities are plotted for each dataset. Low-quality samples were identified using Grubb’s test. Samples that fall on or below the red threshold were excluded from the data repository.

We evaluated each sample using the *IQRray*^23^ software, which produces a quality score for individual samples. Using these metrics, we applied Grubb’s statistical test (*outliers* package^24^) to each dataset, identified poor-quality outliers (Figure 3), and excluded these samples (Table 2 (available online only)). Next we used the *DoppelgangR* package^25^ to identify samples that may have been duplicated inadvertently. We manually reviewed sample pairs that *DoppelgangR* flagged as potential duplicates. We excluded most sample pairs that were flagged (Table 2 (available online only)), even if the clinical annotations for both samples were distinct, under the assumption that these samples had somehow been mislabeled. In GSE46449 (Data Citation 1), many samples were biological replicates; we retained one of each replicate set. GSE5462, GSE19804, and GSE20181 (Data Citation 1) contained samples that had been profiled in a paired manner (e.g., pre- and post-treatment); we retained these pairs of samples.

When transcriptomic data are processed in multiple batches, batch assignments can lead to confounding effects^26^. In the clinical annotations, we identified batch-processing information for datasets GSE25507, GSE37199, GSE39582, and GSE40292 (Data Citation 1). We corrected for batch effects using the ComBat software^27^. The *Biomarker Benchmark* repository contains pre- and post-batch-corrected data. For dataset GSE37199, we identified two variables that could have been used for batch correction ("Centre" and "Plate"). Our repository contains batch-corrected data for both of these batch variables (the default is "Plate").

### Machine-learning analysis

We created a document that illustrates how to programmatically download the data files and perform a simple classification analysis using our data (see https://osf.io/4n62k/). This document is coded for the R statistical package, but similar analyses could be performed using other programming languages.

## Additional information

**How to cite this article**: Golightly N. P. *et al.* Curated compendium of human transcriptional biomarker data. *Sci. Data* 5:180066 doi: 10.1038/sdata.2018.66 (2018).

**Publisher’s note**: Springer Nature remains neutral with regard to jurisdictional claims in published maps and institutional affiliations.

## Supplementary Material



## Figures and Tables

**Table 1:** Overview of data sources used in this study.

**Series ID** a	**Tissue type(s)**	**# Samples**	**Clinical variable(s)**	**# Genes**	**Affymetrix Platform(s)**
GSE1456^28^	Breast cancer	157	Elston grade; overall survival status; overall survival time; relapse status; relapse time	11832	Genome U133A
GSE2109^15^	Breast	263	Age; alcohol consumption; days from diagnosis to excision; ER status; ethnic background; ethnic background; family history of cancer; fibrocystic disease; Her2 status; histology; hormonal therapy duration; mammogram status and findings; metastasis; metastatic sites; multiple tumors; node involvement; oophorectomy status; oral contraceptive use; PR status; prior therapy status; quality metric; relapse time; retreatment states; sex; stage; tobacco use; tumor grade; tumor size	20024	Genome U133 Plus 2.0
GSE2109^15^	Colon	255	Age; alcohol consumption; days from diagnosis to excision; diagnosis method; Dukes’ stage; ethnic background; family history of cancer; histology; metastasis; metastatic sites; multiple tumors; node involvement; primary site; prior screening status; prior therapy status; quality metric; relapse time; retreatment states; sex; stage; symptoms; tobacco use; tumor grade; tumor size	20024	Genome U133 Plus 2.0
GSE2109^15^	Endometrium	51	Age; alcohol consumption; days from diagnosis to excision; ethnic background; family history of cancer; histology; metastasis; metastatic sites; multiple tumors; node involvement; primary site; quality metric; stage; symptoms; tobacco use; tumor grade; tumor size	20024	Genome U133 Plus 2.0
GSE2109^15^	Kidney	209	Age; alcohol consumption; days from diagnosis to excision; ethnic background; family history of cancer; histology; metastasis; metastatic sites; multiple tumors; primary site; prior therapy status; quality metric; relapse time; retreatment states; sex; stage; tobacco use; tumor grade; tumor size	20024	Genome U133 Plus 2.0
GSE2109^15^	Lung	103	Age; alcohol consumption; days from diagnosis to excision; ethnic background; family history of cancer; histology; metastasis; metastatic sites; multiple tumors; node involvement; primary site; prior therapy status; quality metric; relapse time; retreatment states; sex; stage; symptoms; tobacco use; tumor grade; tumor size	20024	Genome U133 Plus 2.0
GSE2109^15^	Ovary	158	Age; alcohol consumption; days from diagnosis to excision; esophagitis reflux history; ethnic background; family history of cancer; fibrocystic disease; histology; mammogram history; metastasis; metastatic sites; multiple tumors; node involvement; node involvement; oophorectomy status; primary site; prior therapy status; quality metric; relapse time; retreatment states; screening history; stage; symptoms; tobacco use; tumor grade; tumor size	20024	Genome U133 Plus 2.0
GSE2109^15^	Prostate	79	Age; alcohol consumption; days from diagnosis to excision; diagnosis method; ethnic background; family history of cancer; Gleason score; histology; metastasis; multiple tumors; node involvement; prior therapy status; prostate-specific antigen (PSA) testing history; PSA finding; quality metric; stage; symptoms; tobacco use; tobacco use; tumor grade; tumor size	20024	Genome U133 Plus 2.0
GSE2109^15^	Uterine	112	Age; alcohol consumption; days from diagnosis to excision; ethnic background; family history of cancer; histology; human papilloma virus diagnosis history; metastasis; metastatic sites; multiple tumors; node involvement; primary site; prior therapy status; quality metric; relapse; stage; symptoms; tobacco use; tumor grade; tumor size	20024	Genome U133 Plus 2.0
GSE4271^29,30^	Glial	100	Age; recurrence status; sex; survival status; survival time; WHO grade	11832	Genome U133A
GSE5460^31^	Breast cancer	127	ER status; Her2 status; histological type; lymphovascular invasion; node status; tumor grade; tumor size	20024	Genome U133 Plus 2.0
GSE5462^32,33^	Breast cancer	52	Treatment history; treatment response	11832	Genome U133A
GSE6532^34^	Breast carcinoma	317	Age; distant metastasis-free survival time/status; ER status; genomic grade index; node involvement; PR status; recurrence-free survival time/status; tumor grade; tumor size	11832	Genome U133A
GSE6532^34^	Breast carcinoma	87	Age; distant metastasis-free survival time/status; ER status; genomic grade index; node involvement; PR status; recurrence-free survival time/status; tumor grade; tumor size	20024	Genome U133 Plus 2.0
GSE10320^35^	Wilms Tumor	144	Relapse	11832	Genome U133A
GSE15296^36^	Peripheral Blood	75	Kidney transplant rejection; subtype	20024	Genome U133 Plus 2.0
GSE19804^37^	Paired tumor and normal tissues	60	Age; tissue type; tumor stage	20024	Genome U133 Plus 2.0
GSE20181^33,38^	Breast cancer	50	Treatment history; treatment response	11832	Genome U133A
GSE20189^39^	Lung adenocarcinoma	162	Case/control status; morphology; smoking status; stage	11832	Genome U133A 2.0
GSE21510^40^	Laser capture microdissection and homogenized tissues (surgically resected material)	104	Metastasis; stage; tissue type	20024	Genome U133 Plus 2.0
GSE25507^41^	Peripheral blood lymphocyte	146	Case/control status (autism); paternal age, maternal age, subject age	20024	Genome U133 Plus 2.0
GSE26682^42–44^	Colorectal tumor	140	Age; microsatellite instability status; sex	11832	Genome U133A
GSE26682^42–44^	Colorectal tumor	160	Age; microsatellite instability status; sex	20024	Genome U133 Plus 2.0
GSE27279^45^	Posterior Fossa Ependymoma	100	Age; sex; tumor location	16632	Exon 1.0 ST
GSE27342^46,47^	Paired gastric tumor and normal tissue	72	Age; sex; stage; tissue type; tumor grade	16632	Exon 1.0 ST
GSE27854^48^	Colorectal tumor	115	Metastasis; stage	20024	Genome U133 Plus 2.0
GSE30219^49^	Lung	293	Age; follow-up time; histology; metastasis; node involvement; relapse status; sex; survival; survival time; tumor size	20024	Genome U133 Plus 2.0
GSE30784^50^	Oral squamous cell carcinoma	229	Age; case/control status; sex	20024	Genome U133 Plus 2.0
GSE32646^51^	Breast	115	Age; ER status (IHC); Her2 status (FISH); histological grade; lymph node involvement; pathologic complete response; PR status (IHC); stage; tumor size	20024	Genome U133 Plus 2.0
GSE37147^52^	Bronchial sample	238	Age; case/control status (COPD); FEV1/FVC score/percentage; inhaled medication status; sex; smoking status; tobacco use	21614	Gene 1.0 ST
GSE37199^53^	Blood sample	94	Disease stage (advanced castration resistant prostate cancer)	20024	Genome U133 Plus 2.0
GSE37745^54^	Non-small cell lung cancer	196	Adjuvant treatment status; age; histology; recurrence time/status; sex; stage; survival time/status; WHO performance status	20024	Genome U133 Plus 2.0
GSE37892^55^	Stage-II colon carcinoma	130	Age; diagnosis history; localisation; stage; time until metastasis	20024	Genome U133 Plus 2.0
GSE38958^56^	Peripheral blood mononuclear cell	115	Age; diagnosis (Idiopathic pulmonary fibrosis); ethnicity; predicted FVC percent; sex	16632	Exon 1.0 ST
GSE39491^57^	Esophageal and gastric samples	120	Tumor cell type	11832	Genome U133 Plus 2.0
GSE39582^58^	Colon cancer	566	Adjuvant chemotherapy; age; BRAF mutation status; chromosome instability status; CIMP status; KRAS mutation status; mismatch repair status; overall survival time/status; recurrence-free survival time/status; sex; stage; TP53 mutation status; tumor location	20024	Genome U133 Plus 2.0
GSE40292^59^	Afferent limb tissue and whole-blood sample	195	Diagnosis; sex	21614	Gene 1.0 ST
GSE43176^60^	Leukemic blast sample	104	Cytogenetics; disease state; FAB stage; KRAS mutation status; NRAS mutation status; subtype	11832	Genome U133A
GSE46449^61^	Peripheral blood leukocyte	53	Age; diagnosis (bipolar disorder)	20024	Genome U133 Plus 2.0
GSE46691^62^	Prostate	545	Gleason score; metastasis	16632	Exon 1.0 ST
GSE46995^63^	Leukocyte	85	Age; disease status (biliary atresia)	21614	Gene 1.0 ST
GSE48391^64^	Breast	81	ER status; gene-expression subtype; Her2 status; recurrence status; survival time/status	20024	Genome U133 Plus 2.0
GSE58697^65^	Desmoid tumor	72	Age; follow-up time; recurrence time; sex; tumor location; tumor size	20024	Genome U133 Plus 2.0
GSE63885^66^	Ovarian cancer surgical sample	101	Adjuvant chemotherapy; BRCA mutation status; clinical status at last follow-up, clinical status after 1st line chemotherapy; disease-free survival; FIGO stage; histopathological type; overall survival; residual tumor size; TP53 accumulation in cancer cells (IHC); TP53 mutation status; TP53 mutation status; tumor grade	20024	Genome U133 Plus 2.0
GSE67784^67^	Peripheral blood sample	309	Sex; V30M mutation status; whether exhibiting symptoms	21614	Gene 1.1 ST

^a^These identifiers represent data series in Gene Expression Omnibus. Some identifiers are listed multiple times; in these cases, we used a subset of the series data (for a specific tissue type or microarray platform).

**Table 2:** *Summary of excluded samples.*

**Series**	**Sample**	**Reason**
GSE15296	GSM382283	Poor Quality
GSE19804	GSM494596	Poor Quality
GSE19804	GSM494654	Poor Quality
GSE19804	GSM494657	Poor Quality
GSE20181	GSM506289	Likely Duplicate
GSE20181	GSM506294	Likely Duplicate
GSE20181	GSM506304	Likely Duplicate
GSE20181	GSM125198	Likely Duplicate
GSE20181	GSM125210	Likely Duplicate
GSE20181	GSM125230	Likely Duplicate
GSE2109_Breast	GSM53059	Likely Duplicate
GSE2109_Breast	GSM53027	Likely Duplicate
GSE2109_Colon	GSM89040	Likely Duplicate
GSE2109_Colon	GSM152664	Likely Duplicate
GSE2109_Colon	GSM152632	Likely Duplicate
GSE2109_Colon	GSM179922	Likely Duplicate
GSE2109_Colon	GSM89044	Likely Duplicate
GSE2109_Colon	GSM152666	Likely Duplicate
GSE2109_Colon	GSM179820	Likely Duplicate
GSE2109_Colon	GSM179924	Likely Duplicate
GSE2109_Lung	GSM203652	Poor Quality
GSE2109_Ovary	GSM76554	Likely Duplicate
GSE2109_Ovary	GSM203725	Likely Duplicate
GSE2109_Ovary	GSM76567	Likely Duplicate
GSE2109_Ovary	GSM231913	Likely Duplicate
GSE2109_Ovary	GSM46839	Poor Quality
GSE2109_Prostate	GSM179790	Likely Duplicate
GSE2109_Prostate	GSM179843	Likely Duplicate
GSE2109_Prostate	GSM179903	Likely Duplicate
GSE25507	GSM627091	Likely Duplicate
GSE25507	GSM627087	Likely Duplicate
GSE25507	GSM627096	Likely Duplicate
GSE25507	GSM627078	Likely Duplicate
GSE25507	GSM627085	Likely Duplicate
GSE25507	GSM627196	Likely Duplicate
GSE25507	GSM627153	Likely Duplicate
GSE25507	GSM627180	Likely Duplicate
GSE25507	GSM627099	Likely Duplicate
GSE25507	GSM627115	Likely Duplicate
GSE25507	GSM627118	Likely Duplicate
GSE25507	GSM627124	Likely Duplicate
GSE25507	GSM627154	Likely Duplicate
GSE25507	GSM627204	Likely Duplicate
GSE25507	GSM627209	Likely Duplicate
GSE25507	GSM627215	Likely Duplicate
GSE26682	GSM656833	Likely Duplicate
GSE26682	GSM656770	Likely Duplicate
GSE26682_U133PLUS2	GSM656860	Poor Quality
GSE26682_U133PLUS2	GSM656613	Poor Quality
GSE26682_U133PLUS2	GSM656839	Poor Quality
GSE26682_U133PLUS2	GSM656721	Poor Quality
GSE27342	GSM675945	Likely Duplicate
GSE27342	GSM675947	Likely Duplicate
GSE27342	GSM675933	Likely Duplicate
GSE27342	GSM675935	Likely Duplicate
GSE27342	GSM676040	Poor Quality
GSE27342	GSM687519	Poor Quality
GSE27854	GSM687525	Poor Quality
GSE30219	GSM748210	Likely Duplicate
GSE30219	GSM748212	Likely Duplicate
GSE30219	GSM748218	Likely Duplicate
GSE30219	GSM748219	Likely Duplicate
GSE30219	GSM748255	Poor Quality
GSE30219	GSM748247	Poor Quality
GSE30219	GSM748057	Poor Quality
GSE30219	GSM748266	Poor Quality
GSE30784	GSM764928	Likely Duplicate
GSE30784	GSM764930	Likely Duplicate
GSE30784	GSM764904	Poor Quality
GSE30784	GSM764970	Poor Quality
GSE32646	GSM809214	Likely Duplicate
GSE32646	GSM809248	Likely Duplicate
GSE32646	GSM809251	Likely Duplicate
GSE32646	GSM809254	Likely Duplicate
GSE37147	GSM912230	Likely Duplicate
GSE37147	GSM912296	Likely Duplicate
GSE37147	GSM912296	Likely Duplicate
GSE37147	GSM912305	Likely Duplicate
GSE37147	GSM912291	Likely Duplicate
GSE37147	GSM912296	Likely Duplicate
GSE37147	GSM912305	Likely Duplicate
GSE37147	GSM912342	Likely Duplicate
GSE37147	GSM912273	Likely Duplicate
GSE37147	GSM912305	Likely Duplicate
GSE37147	GSM912342	Likely Duplicate
GSE37147	GSM912342	Likely Duplicate
GSE37147	GSM912348	Likely Duplicate
GSE37147	GSM912376	Likely Duplicate
GSE37147	GSM912376	Likely Duplicate
GSE37147	GSM912376	Likely Duplicate
GSE37147	GSM912463	Poor Quality
GSE37147	GSM912197	Poor Quality
GSE37147	GSM912300	Poor Quality
GSE37199	GSM913439	Poor Quality
GSE37745	GSM1019319	Likely Duplicate
GSE37745	GSM1019246	Likely Duplicate
GSE37745	GSM1019325	Likely Duplicate
GSE37745	GSM1019247	Likely Duplicate
GSE37745	GSM1019194	Poor Quality
GSE37745	GSM1019195	Poor Quality
GSE37745	GSM1019176	Poor Quality
GSE37745	GSM1019192	Poor Quality
GSE37745	GSM1019232	Poor Quality
GSE37892	GSM929512	Poor Quality
GSE39491	GSM970152	Poor Quality
GSE39582	GSM972249	Likely Duplicate
GSE39582	GSM972472	Likely Duplicate
GSE39582	GSM972243	Likely Duplicate
GSE39582	GSM972044	Likely Duplicate
GSE39582	GSM972091	Likely Duplicate
GSE39582	GSM972090	Likely Duplicate
GSE39582	GSM972245	Likely Duplicate
GSE39582	GSM972473	Likely Duplicate
GSE39582	GSM972515	Likely Duplicate
GSE39582	GSM972248	Likely Duplicate
GSE43176	GSM1057835	Poor Quality
GSE46449	GSM1130404	Likely Duplicate
GSE46449	GSM1130406	Likely Duplicate
GSE46449	GSM1130413	Likely Duplicate
GSE46449	GSM1130417	Likely Duplicate
GSE46449	GSM1130426	Likely Duplicate
GSE46449	GSM1130428	Likely Duplicate
GSE46449	GSM1130430	Likely Duplicate
GSE46449	GSM1130434	Likely Duplicate
GSE46449	GSM1130436	Likely Duplicate
GSE46449	GSM1130468	Likely Duplicate
GSE46449	GSM1130471	Likely Duplicate
GSE46449	GSM1130483	Likely Duplicate
GSE48390	GSM1176924	Poor Quality
GSE48390	GSM125120	Poor Quality
GSE5462	GSM125123	Likely Duplicate
GSE5462	GSM125125	Likely Duplicate
GSE58697	GSM1417097	Poor Quality
GSE63885	GSM1559328	Likely Duplicate
GSE63885	GSM1559360	Likely Duplicate
GSE63885	GSM1559385	Likely Duplicate
GSE63885	GSM1559370	Likely Duplicate
GSE63885	GSM1559375	Likely Duplicate
GSE63885	GSM1559386	Likely Duplicate
GSE63885	GSM1559361	Poor Quality
GSE6532_U133PLUS2	GSM151294	Poor Quality
GSE6532_U133PLUS2	GSM151280	Poor Quality
We excluded samples that did not pass our quality-control criteria or that appeared to be duplicated. The Gene Expression Omnibus series and sample identifiers are listed, along with the reason we excluded each sample.		
